# Exosome-mediated delivery of siRNA molecules in cancer therapy: triumphs and challenges

**DOI:** 10.3389/fmolb.2024.1447953

**Published:** 2024-09-17

**Authors:** Philemon Ubanako, Sheefa Mirza, Paul Ruff, Clement Penny

**Affiliations:** Department of Internal Medicine, Faculty of Health Sciences, University of the Witwatersrand, Johannesburg, South Africa

**Keywords:** cancer, exosomes, siRNA, gene expression, drug delivery, therapeutics

## Abstract

The discovery of novel and innovative therapeutic strategies for cancer treatment and management remains a major global challenge. Exosomes are endogenous nanoscale extracellular vesicles that have garnered increasing attention as innovative vehicles for advanced drug delivery and targeted therapy. The attractive physicochemical and biological properties of exosomes, including increased permeability, biocompatibility, extended half-life in circulation, reduced toxicity and immunogenicity, and multiple functionalization strategies, have made them preferred drug delivery vehicles in cancer and other diseases. Small interfering RNAs (siRNAs) are remarkably able to target any known gene: an attribute harnessed to knock down cancer-associated genes as a viable strategy in cancer management. Extensive research on exosome-mediated delivery of siRNAs for targeting diverse types of cancer has yielded promising results for anticancer therapy, with some formulations progressing through clinical trials. This review catalogs recent advances in exosome-mediated siRNA delivery in several types of cancer, including the manifold benefits and minimal drawbacks of such innovative delivery systems. Additionally, we have highlighted the potential of plant-derived exosomes as innovative drug delivery systems for cancer treatment, offering numerous advantages such as biocompatibility, scalability, and reduced toxicity compared to traditional methods. These exosomes, with their unique characteristics and potential for effective siRNA delivery, represent a significant advancement in nanomedicine and cancer therapeutics. Further exploration of their manufacturing processes and biological mechanisms could significantly advance natural medicine and enhance the efficacy of exosome-based therapies.

## 1 Introduction

Cancer is a complex group of diseases that results from the multistage development of mutations of cancer-associated genes, leading to deregulated cell proliferation and potential for metastasis and invasion (Stratton et al., 2009). A major global challenge is finding novel and innovative therapeutic strategies for cancer treatment and management. Several conventional chemotherapeutic agents display low aqueous solubility and stability, rapid metabolism, and indiscriminate drug distribution. These lead to low drug bioavailability at the target site, poor efficacy, dose-limiting toxicity, and debilitating side effects (Iwamoto, 2013; Yao et al., 2020). This has prompted the development of targeted or non-targeted lipid- or polymer-based nanoparticle drug delivery systems (Yang and Wu, 2018). Nanoscale drug delivery tools have garnered considerable prominence due to improved pharmacokinetic and safety characteristics and increased bioavailability and efficacy of the loaded compounds (Liu et al., 2016; Yang and Wu, 2018).

A few anticancer drug-nanoparticle formulations have been approved for clinical use, and some have progressed to clinical trials. For example, Abraxane is a paclitaxel-loaded albumin-bound nano formulation approved by the FDA in 2005 for treating metastatic breast cancer (Desai et al., 2006). Abraxane showed enhanced tumor penetration and anti-tumoral activity compared with an equal dose of plain paclitaxel (Desai et al., 2006). Moreover, studies in clinical trials and *in vivo* mouse models have demonstrated that paclitaxel liposomal formulations significantly outperform conventional paclitaxel (Koudelka and Turánek, 2012). Other nanoparticle-based formulations include Onivyde, a liposomal formulation of irinotecan approved by the FDA in 2015 and 2024 for the treatment of colorectal and metastatic pancreatic cancers, respectively (Chen et al., 2024; Passero et al., 2016; Zhang, 2016). Vyxeos is also a lipid nanoparticle formulation of cytarabine/daunorubicin approved in 2017 and 2018 for treating acute myeloid leukemia (Krauss et al., 2019).

Despite substantial research in nanoparticle-based drug delivery systems in the past five decades, their clinical translation has been minimal. Critical challenges experienced by synthetic nanoparticle delivery systems include reduced efficiency in reaching target tissue, high toxicity and immunogenicity, and reduced half-life in circulation (Vader et al., 2016). These limitations have prompted researchers to investigate more efficient and biocompatible nano-range drug carriers, such as exosomes, for cancer therapy. Exosomes are nanoscale endogenous extracellular vesicles that have gained increasing attention as novel and innovative structures for advanced drug delivery and targeted therapy. The superior physicochemical and biological properties of exosomes, which include increased permeability, biocompatibility, and half-life in circulation, coupled with reduced toxicity and immunogenicity compared to synthetic nanoparticles, have made them preferred drug delivery vehicles in cancer and other diseases (Luan et al., 2017).

The selective knockdown of cancer-related genes using short interfering RNA (siRNA) molecules has proven effective in suppressing cell proliferation and metastasis *in vitro* and *in vivo*. However, the intracellular delivery of siRNA has always been a significant challenge, which hampers its efficacy (Tatiparti et al., 2017). Nanoparticle delivery systems such as liposomes, polymeric nanoparticles, and dendrimers have been reported to deliver siRNA to target cancer and other diseases (Grodzicka et al., 2024; Tatiparti et al., 2017; Zhang et al., 2023). Nevertheless, these formulations face challenges in clinical translation such as off-target effects, poor stability and safety. This necessitates the need to develop efficient and biocompatible nanosystems for targeted siRNA delivery (Kesharwani et al., 2012). Exosomes are effective nanosystems for siRNA delivery, demonstrating superior gene knockdown and associated anticancer therapeutic effects (Lin et al., 2021; Lin et al., 2022; Zhao et al., 2020). This review explores exosome-mediated delivery of siRNAs targeting various genes in diverse types of cancer. It provides advantages, challenges, and avenues for improving exosome-based therapies in cancer for better disease management.

### 1.1 Exosome biology, biogenesis, and secretion

A defining hallmark of multicellular organisms is their ability to communicate intercellularly by cell-to-cell contact, secretion of bioactive molecules, or the release and uptake of extracellular vesicles. These bioactive molecules include small RNA molecules such as mRNA, miRNA, long, non-coding RNA, circular RNA, DNA, and proteins (Dai et al., 2020). Because exosomes naturally transport diverse types of RNA molecules between cells, they can be exploited to transport and deliver therapeutic siRNA molecules to cancer cells, which target aberrantly expressed cancer-associated genes (Ruivo et al., 2017).

Extracellular vesicles comprise microvesicles, apoptotic bodies, and exosomes; distinguished primarily by their size, method of biogenesis, and composition. MVs, which have a diameter of 100–1,000 nm and are larger than exosomes, are produced by evagination and fission of the plasma membrane. Their main contents are cytosolic material, which includes lipids, proteins, nucleic acids, and metabolites (Buzas, 2023; Jeppesen et al., 2019). The largest of the three EV categories, apoptotic bodies, range usually between 1,000 and 5,000 nm in size. They are produced during apoptosis when cells disintegrate into smaller fragments and become packaged into membrane-bound vesicles. Apoptotic bodies comprise DNA fragments, histones, chromatin remnants, cytosolic components, and damaged proteins. Phagocytic cells usually remove apoptotic bodies (Buzas, 2023; Dixson et al., 2023). According to the Minimal Information for Studies of Extracellular Vesicles (MISEV 2023) (Welsh et al., 2024), and other articles (Matsumoto et al., 2020; Yekula et al., 2020), extracellular vesicles that range between 30 and 250 nm in diameter are “small EVs” (sEVs). sEVs are naturally secreted by endogenous cells and are primarily involved in intercellular communication through the cellular exchange of nucleic acids, lipids, proteins, and metabolites (Welsh et al., 2024). However, since we refer to numerous studies throughout this manuscript that used the term “exosome,” we have maintained the use of the term to denote these sEVs for simplicity. Hence, exosomes are nanoscale (30–250 nm), lipid bilayer-enclosed, extracellular vesicles of endocytic origin released from most cell types, including fibroblasts, mesenchymal, primary, immune, and cancer cells, and are critical for cell-cell communication (Li P. et al., 2017; McLellan, 2009; Welsh et al., 2024; Yan and Jiang, 2020; Zhang et al., 2014).

**FIGURE 1:**
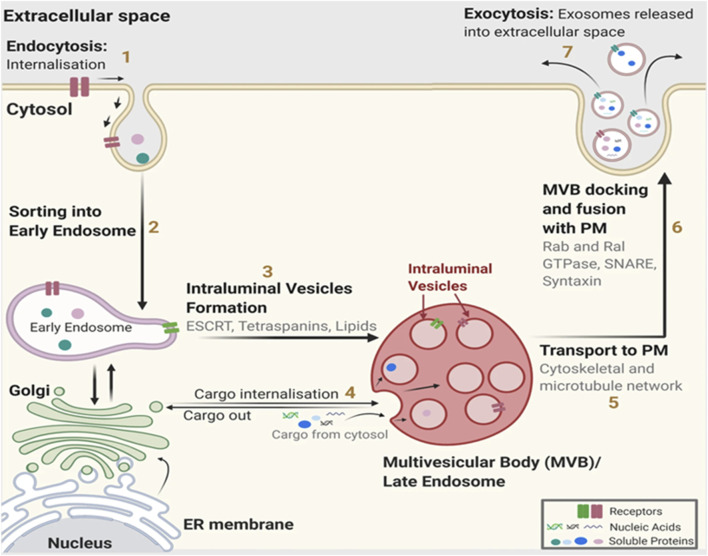
Exosome biogenesis. (1) The relevant cargoes are internalized by endocytosis. (2) Internalized cargoes are sorted into early endosomes. (3) The early endosomes mature into late endosomes, also known as multivesicular bodies (MVB). Late endosomes/MVBs are specialized endosomal compartments containing intraluminal vesicles (ILVs) that sequester proteins, lipids, cytosolic compartments, and potential exosome cargoes. (4) Cargoes are also delivered from the trans-Golgi network and possibly from the cytosol. (5) Late endosomes containing exosome cargoes get (5) transported to the plasma membrane, (6) fuse with the cell surface and, (7) the ILVs are secreted as exosomes. PM: plasma membrane, ER: endoplasmic reticulum. (Figure used with permission from Gurung et al. (2021)).

Exosome biogenesis begins by invaginating the plasma membrane to produce early endosomes. The early endosomes mature into late endosomes, during which the endosome membrane invaginates to produce intraluminal vesicles (ILVs) in the lumen of the organelles, which encapsulates biomolecules (McAndrews and Kalluri, 2019). Multivesicular bodies (MVBs) are late endosomes containing scores of ILVs that are subsequently: i) transported to Golgi bodies for endosome recycling, ii) delivered to lysosomes for breakdown of all contained material, or iii) fuse with the plasma membrane and release exosomes extracellularly (Williams and Urbé, 2007). Mechanistically, exosome biogenesis and their eventual extracellular release require the formation of an endosomal sorting complex required for transport (ESCRT) proteins and associated factors. However, ESCRT-independent mechanisms have been described previously (McAndrews and Kalluri, 2019). Exosome biogenesis and secretion are illustrated in Figure 1.

Cell-cell communication mediated by exosomes can occur via three strategies: First, an intracellular signaling cascade can be triggered when exosomal membrane proteins contact cell membrane proteins. Second, exosomal membrane-cell fusion leads to endocytosis and release of exosomal cargo into recipient cells, which influences cell phenotype. Third, recipient cells could directly phagocytose cargo-laden exosomes, releasing the exosomal contents intracellularly (Barros et al., 2018).

Exosomes were first demonstrated to transport functional small RNA molecules such as mRNA and miRNA between different cells, proving they were not only inert, waste-containing extracellular vesicles (Valadi et al., 2007). Shortly after this, Skog et al. (2008) showed that exosomes derived from glioblastoma cells could be detected in serum and contained angiogenic proteins and mRNA that could be taken up by other host cells, including endothelial and brain cells. They further showed that these biochemical messages shuttled by exosomes could induce cell proliferation in a glioma cell line (Skog et al., 2008). These seminal findings established the role of exosomes as mediators of intercellular communication.

**FIGURE 2:**
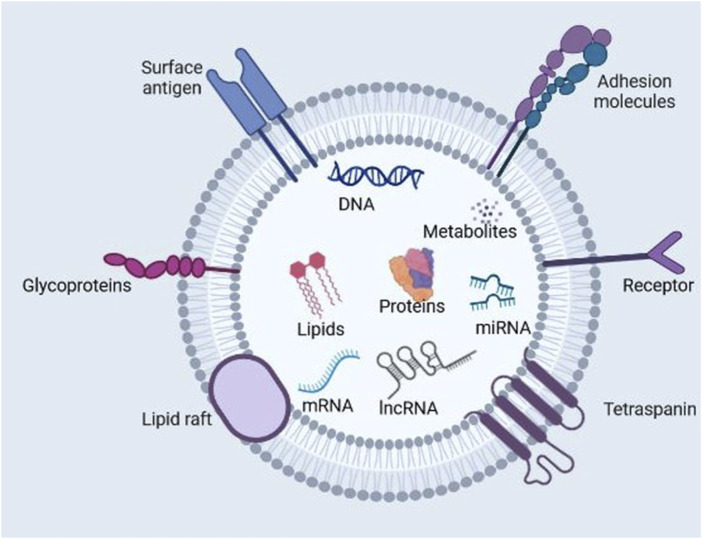
Exosome composition. Exosomes are nano-sized extracellular vesicles produced naturally by all cell types. They contain proteins, nucleic acids, lipids, and metabolites. Exosomes function as intermediaries of intercellular communication and influence different facets of cell biology in disease and normal physiology. (Figure created with Biorender).

The biological effect conferred by exosomes on nearby or distant cells is based on their bioactive cargo, such as mRNAs, miRNAs, proteins, lipids, etc., which can activate specific cell signaling cascades, thereby inducing pathological or physiological responses (Figure 2). According to Exocarta (http://www.exocarta.org/), a database dedicated to research on exosomal contents, up to 286 studies have identified 41,860 proteins, 7,540 RNAs, and 1,116 lipids. The innate ability of exosomes to ferry bioactive substances throughout the body has been exploited for drug delivery applications (Haney et al., 2015; Kamerkar et al., 2017; Li W. et al., 2017; Purushothaman et al., 2016).

**FIGURE 3:**
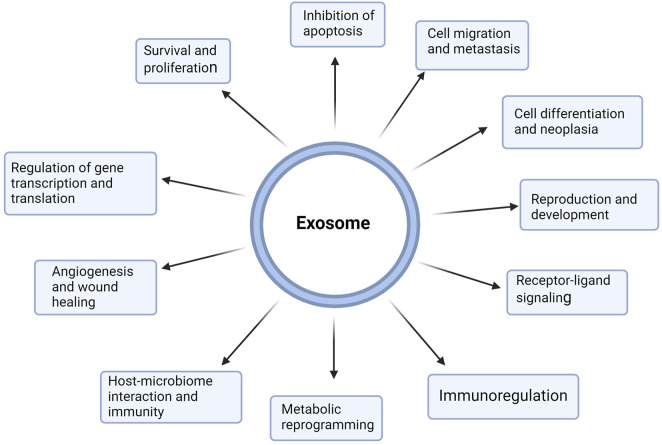
Multifunctional roles of exosomes in health and disease. Exosomes are nanoscale extracellular vesicles produced by all cells and contain metabolites, proteins, lipids, and nucleic acids, which are pivotal for cell-cell communication. Exosomes mediate near and far intercellular communication in health and disease, influencing different facets of cell biology. Exosome-delivered proteins, metabolites, nucleic acids, and lipids significantly influence the biological response of recipient cells. Such exosome-mediated cellular responses may promote or inhibit the development of diseases such as cancer. Exosomes participate in proliferation, metastasis, immunoregulation, apoptosis, cell differentiation, metabolic reprogramming, cell signaling, gene regulation, and reproduction. (Figure created with Biorender).

Exosomes have gained increasing attention as nanocarriers for cancer therapeutics owing to their increased permeability, biocompatibility, extended half-life in circulation, and reduced toxicity and immunogenicity (Vader et al., 2016). The enhanced biocompatibility and cellular uptake of exosomes compared with synthetic nanoparticle delivery systems can be attributed to the surface expression of membrane proteins such as the tetraspanins (e.g., CD9, CD63, and CD81), fibronectin integrins, immunoglobulins, etc. which are modifiable based on target cells (Mulcahy et al., 2014; Purushothaman et al., 2016). Soltani et al. (2015) also showed that exosomes display enhanced stability in bodily fluids compared to liposomes with similar characteristics. Liposomes, for example, are rapidly phagocytosed by the cells in the reticuloendothelial system or macrophages (Soltani et al., 2015). Exosomes have multiple functions in health and disease, including modulating the immune responses, metabolic reprogramming, survival and proliferation, gene regulation, angiogenesis, metastasis, receptor-ligand signaling, etc. (Kalluri and LeBleu, 2020) (Figure 3).

### 1.2 Advantages of exosomes for drug delivery

Exosomes possess several attractive attributes which are explored in drug delivery. Exosomes’ endogenous origin and similarity to cell membranes render them more biocompatible. Several studies have proposed that exosomes can evade recognition and subsequent destruction by immune system cells, thereby improving their half-life in circulation, an attribute critical for efficient drug delivery. Their ability to evade immunosurveillance mechanisms renders them as immune-compatible drug delivery systems (Gutiérrez-Vázquez et al., 2013; Haney et al., 2015; Whiteside, 2013). The slightly negative zeta potential characteristic of exosomes also facilitates prolonged circulation in biological fluids (Malhotra et al., 2016). Moreover, some exosomes have shown superior delivery of drugs and siRNA molecules compared to regularly used nanosystems such as liposomes and polymeric nanoparticles (Zheng et al., 2019).

Exosome-based drug delivery can improve therapeutic effects in animal tumor models compared to free drugs. In one study, paclitaxel, a chemotherapeutic drug that inhibits microtubules and induces cell cycle arrest and apoptosis, was successfully incorporated into exosomes using sonication. *In vitro*, these drug-laden exosomes were up to 50 times more cytotoxic than free paclitaxel, to paclitaxel-resistant cancer cells (Batrakova and Kim, 2015). The small size of exosomes enables them to penetrate biological barriers such as the blood-brain barrier (Alvarez-Erviti et al., 2011; Vader et al., 2016) and improve the stability of drugs with low stability, such as curcumin (Sun et al., 2010). Furthermore, exosomes have transmembrane and membrane-bound peptides/proteins that promote endocytosis and, as a result, facilitate the transfer of their cargo into target cells (Kamerkar et al., 2017). Exosome-based drug delivery platforms can significantly minimize side effects compared to free chemotherapeutic drugs. Exosomes loaded with various chemotherapeutic drugs were proven to deliver the drug effectively to mouse tumor sites and suppress tumor development with fewer side effects when compared to the free drug (Batrakova and Kim, 2015). These attractive attributes are vital for successfully applying exosome-based drug delivery systems.

### 1.3 Isolation and characterization of exosomes

Several methods, such as ultracentrifugation, size exclusion chromatography, ultrafiltration, and immunocapture, have been devised to isolate and purify exosomes (Lane et al., 2017; Li P. et al., 2017; Sidhom et al., 2020). These methods have harnessed the biological, chemical, and physical properties of exosomes for their effective isolation and purification. Differential ultracentrifugation is currently the most extensively used method for isolating exosomes, as it has proven effective in several research studies (Jeppesen et al., 2014; Lobb et al., 2015). Exosomes can be detected using transmission electron microscopy (TEM), scanning electron microscopy (SEM), and nanoparticle tracking analysis (NTA). Canonical exosomal markers, such as some tetraspanin proteins (CD9, CD63, and CD81), can also be detected using immunoblotting and antibody-based magnetic bead flow cytometry assays (Soo et al., 2012; Wu et al., 2015).

### 1.4 Engineering exosomes for targeted anticancer therapy

Exosomes, being natural cell-cell transporters, easily evade immune system clearance strategies. Although exosomes are cellularly manufactured under natural conditions, they can be further engineered artificially, particularly for targeted therapeutic applications. The native structure and composition of exosomes confer biocompatible features essential for clinical applications. However, appropriate modifications are necessary to enhance their efficacy and stability as therapeutic or diagnostic tools (Luan et al., 2017).

Passive targeting of drug-loaded exosomes or other nanoparticle vehicles to tumor tissues capitalizes primarily on the vulnerabilities of various components of the tumor environment distinct from normal tissues. These include the enhanced permeability retention (EPR) phenomenon. The EPR effect is characterized by enhanced vascular permeability and compromised lymphatic drainage of cancer cells, resulting in the selective accumulation of nanoparticles such as exosomes in tumor tissue (Sano et al., 2013).

Active cancer targeting by nano vectors to malignant cells involves direct interactions between targeting ligands and cancer-associated receptors (Kamaly et al., 2012; Yao et al., 2020). Exosomes can be engineered to express specific ligands on their surface, targeting overexpressed receptors exclusively on the surface of malignant and not healthy cells. The engineered exosomal ligands can then interact with their cognate receptors on the surface of malignant cells, thereby triggering receptor-mediated endocytosis and leading to the release of therapeutic drugs by the internalized exosome (Farokhzad and Langer, 2009). Exosomes can be engineered using ligands such as tumor–homing and penetrating peptide (tLyp-1), lysosomal associated membrane protein 2 (Lamp2b), Internalizing arginine-glycine-aspartic acid (iRGD), and PEG-PEI (copolymer of cationic poly (ethylene imine) (PEI) and polyethylene glycol (PEG) to improve their therapeutic targeting ability (Alvarez-Erviti et al., 2011; Bai et al., 2020; Lin et al., 2021; Lin et al., 2022; Zhou et al., 2019).

### 1.5 siRNA biology and mechanism of action

siRNAs possess immense therapeutic potential due to their ability to target the mRNA of any cancer-associated gene selectively. This selective gene knockdown ability of siRNAs is especially crucial for biochemically intractable drug targets in oncology, previously dubbed as ‘undruggable’ or better termed ‘difficult to drug’ targets (Dang et al., 2017; Setten et al., 2019). mRNA molecules contain vital information that regulates a plethora of biological processes with profound clinical implications. The role of a specific gene can be confirmed by suppressing the protein of interest and studying the corresponding phenotypic changes using biochemical, histological, and pharmacological investigations, among other methods (Xie et al., 2006). mRNA transcripts derived from oncogenes and other cancer-associated genes can be effectively suppressed using RNA interference mechanisms such as small interfering RNA (siRNA), thereby curbing cancer progression (Reischl and Zimmer, 2009; Singh et al., 2018). This has resulted in significantly intensified research efforts to formulate siRNA-based anticancer therapeutic strategies (Davis, 2009; Davis et al., 2010; Dong et al., 2018; Wang X. et al., 2020; Zhao et al., 2020).

**FIGURE 4:**
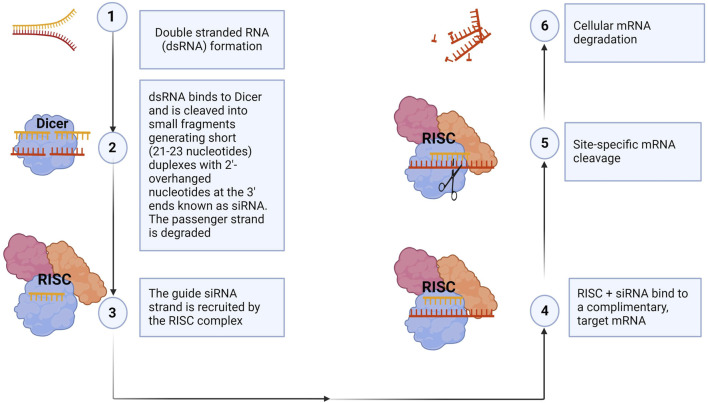
siRNA mechanism of action. The siRNA machinery is an evolutionarily conserved mechanism for the suppression of gene expression. In summary, dicer, a ribonuclease, breaks down long double-stranded RNA, generating short siRNA duplexes (1, 2). siRNAs consist of two overhanging nucleotides and a short 20–24-bp double-stranded RNA (dsRNA) with phosphorylated 5′ and hydroxylated 3′ ends. The siRNA is incorporated into the RNA-induced silencing complex (RISC), where Argonaut 2 protein interacts, degrading the passenger strand (3). The RISC guides the guide strand to the complementary mRNA, causing catalytic cleavage and degradation, ultimately suppressing gene expression (4–6). Synthetic siRNAs can also be transfected into cells to knock down, in principle, any gene of interest with a complementary sequence, thereby validating gene function. (Figure created with Biorender).

siRNA is a small RNA molecule with a length of 21–23 nucleotide bases that post-transcriptionally downregulates a target gene expression level by mRNA degradation (Halbur et al., 2019; Wang et al., 2017). The mechanism of siRNA-mediated gene knockdown was elucidated primarily by the Nobel Prize-winning work of Fire and Mello, who discovered that exogenous double-stranded RNA could silence specific genes in the roundworm *Caenorhabditis elegans* (Fire et al., 1998). This groundbreaking discovery can be replicated in virtually all species, from flies to humans. In summary, a specific ribonuclease, dicer, binds to a double-stranded RNA molecule and cleaves it, generating short (21–23 nucleotide bases) duplexes with 2-overhanged nucleotides at the 3′-ends known as siRNA. Next, the siRNA becomes incorporated into a multiprotein complex called the RNA-induced silencing complex (RISC). The siRNA duplex comprises a sense (passenger strand) and an antisense strand (guide strand). In the RISC, the Argonaut 2 protein component interacts with the siRNA duplex, unwinding it and degrading the passenger strand. The guide strand guides the RISC complex to the complementary mRNA. This results in the catalytic cleavage and degradation of the target mRNA by its endonuclease activity and eventual suppression of gene expression (Birmingham et al., 2007; Fakhr et al., 2016; Fire et al., 1998). This mechanism is illustrated in Figure 4.

Although siRNA-mediated gene knockdown can effectively suppress cancer phenotypes, the technology is fraught with several drawbacks that have impeded the clinical translation efforts of siRNA therapeutics. These challenges include difficulties penetrating the cell membrane, siRNA entrapment by the lysosome, vulnerability to nuclease degradation, off-target effects, possible immunogenicity, and instability in serum (Singh et al., 2018). The most pressing concern facing siRNA delivery is their anionic, hydrophilic properties preventing them from penetrating hydrophobic cell membranes (Oh and Park, 2009). Moreover, synthetic siRNAs can invoke an immune response, especially if multiple dosing is required (O’Loughlin et al., 2012). These challenges have spurred diverse methods of formulating siRNA for targeted delivery. Viral vectors, liposomes, and synthetic polymeric nano-formulations are the most widely explored delivery vehicles for siRNA for therapeutic applications. Ultimately, efficient delivery systems are needed to protect siRNAs from endonuclease degradation and deliver them intracellularly without inciting any adverse effects (Subhan and Torchilin, 2019). Several research studies have reported varying degrees of efficacy of these systems for siRNA delivery in cancer. Moreover, chemical modifications such as 2′-O-methylation of the guide strand of siRNA, phosphate, ribose, and base modifications have been used to improve siRNA delivery and mitigate the off-target effects of siRNAs without compromising the silencing activity of siRNAs on target genes (Hu et al., 2020; Watts et al., 2008).

### 1.6 Benefits of siRNA delivery for cancer therapy

siRNAs are double-stranded, non-coding RNAs that unwind into single strands (ssRNA), bind to their target mRNA, and activate a cascade of events that culminate in the catalytic cleavage and subsequent degradation of their cognate mRNA. This leads to a post-translational inhibition or knockdown of gene expression (Fire et al., 1998). Pioneering work demonstrating the power of siRNA molecules to silence genes in mammalian cells was done by Elbashir and colleagues in 2001. In this study, the authors demonstrated that 21-nucleotide siRNA duplexes decrease endogenous and exogenous gene expression in various mammalian cell lines, such as HEK293 and HeLa cells (Elbashir et al., 2001). Since then, many studies have explored using siRNA to target cancer-associated genes as an anticancer strategy. For example, virtually all types of cancer (regarding tissue of origin) have been targeted with siRNAs, showing some degree of success. These include lung, breast, thyroid, brain, cervical, skin (melanoma), prostate, and liver cancer. Several clinical trial studies using RNA interference (RNAi) drug formulations designed to knock down the expression of cancer-related genes have shown encouraging therapeutic responses in cancer patients (Table 1). These trials demonstrate two principal triumphs: effective delivery of siRNAs to tumors and selective knockdown of gene expression. These trials include the knockdown of VEGF, kinesin spindle protein (KSP), and PLK-1 (Cervantes et al., 2011; Demeure et al., 2016).

**TABLE 1:** siRNA drug formulations in clinical trials. Adapted from Jain et al. (2023).

Type of cancer	Drug	Delivery system	Target	Delivery route	Phase	Stage	Clinical trial ID
Liver cancer	ALN-VSP02	Lipid NPs	VEGF and KSP	IV	Phase I	Completed	NCT00882180
Liver cancer	TKM 080,301	Lipid NPs	Polo-kinase-1	IV	Phase I and II		NCT02191878
Advanced solid tumors	TKM 080,301	Lipid NPs	PLK-1	IV	Phase I and II	Completed	NCT01262235
Primary or secondary liver cancer	TKM 080,301	Lipid NPs	PLK-1	IV	Phase I	Completed	NCT01437007
Advanced solid tumors with liver metastases	EZN-2968	Locked nucleic acid	HIF-1	IV	Phase I	Completed	NCT01120288
Advanced solid tumors	Atu027	Lipoplex-siRNA	PKN3	IV	Phase I	Completed	NCT00938574
Pancreatic cancer	siG12D LODER	Biodegradable polymeric matrix	KRAS	Intratumoral administration	Phase I	Completed	NCT01188785
Advanced malignant solid tumors	siRNA-EphA2-DOPC	Liposome	EphA2	IV	Phase I	Active, not recruiting	NCT01591356
Solid tumor	CALAA-01	Cyclodextrin NPs	RRM2	IV	Phase I	Terminated	NCT00689065
Pancreatic cancer	KRAS G12D siRNA	Exosomes	KRAS G12D	IV	Phase I	Active, not recruiting	NCT03608631
Metastatic melanoma	Proteasome siRNA and tumor antigen RNA-transfected dendritic cells	Dendritic Cell	LMP2, LMP7, and MECL1	Intradermal Injection	Phase I	Completed	NCT00672542
Myeloid leukaemia	SV40 vectors carrying siRNA	Viral Vector	BCR-ABL		Phase I	Completed	NCT002647

Owing to the broad knowledge of the human genome, most protein-coding genes have been deciphered and appropriately annotated (Nurk et al., 2021). Hence, the design and synthesis of a complimentary siRNA molecule after identifying the target mRNA becomes simple and efficient, only requiring reliable bioinformatic tools and nucleic acid synthesis procedures. Contrarily, most traditional small molecule therapeutics work at the protein level, necessitating a higher degree of structural accuracy and, as a result, entail a more challenging and complicated development process (Zhang et al., 2021). However, due to several reported challenges such as poor delivery efficiency, toxicity, and immunogenicity, only a few RNAi delivery platforms involving viral vectors or nanoparticles have progressed to clinical trials (Table 1).

Viral vector-based small RNA delivery vehicles pose a risk of insertional oncogenic transformation, inactivation of tumor suppressor genes, low biocompatibility, and high cost of production (Anson, 2004; Nayerossadat et al., 2012). Synthetic polymeric nanoparticles or liposomal systems exhibit poor biocompatibility and delivery efficiency.

### 1.7 Exosome-mediated siRNA delivery in cancer

Unprotected siRNA cannot passively diffuse through the anionic cell membrane due to its large size, high molecular weight, and negative phosphate group charges. It is crucial to comprehend how nanomedicine is delivered *in vivo*. For siRNA-based cancer gene therapy, an intravenously administered therapeutic siRNA must be stable in the blood circulation, accumulate in tumor tissues, be taken up by tumor cells, and identify and degrade target mRNA in the cytoplasm. Although a few RNAi-based therapeutics have been successful in clinical trials, several challenges have impeded their clinical translation. These challenges include the efficiency of siRNA delivery to tumors, the choice of target mRNA, and the biocompatibility or safety of the formulations (Wu et al., 2014).

Pioneering research by Alvarez-Erviti and colleagues in 2011 demonstrated that brain-targeting siRNA-loaded exosomes could be delivered effectively into a mouse brain and induce a corresponding targeted gene knockdown. In this seminal study, dendritic cells harvested from mice were transfected to express the Rabies virus glycoprotein (RVG) coupled with Lamp2b, an exosomal membrane protein. RVG is a neuronal targeting peptide that binds to acetylcholine receptors on neurons. The transfected dendritic cells expressed Lamp2b-RVG, which was then incorporated into the excreted exosomes. Electroporation was used to load the purified exosomes with siRNA molecules against BACE1, a crucial gene in Alzheimer’s disease pathogenesis. The authors showed a 60% downregulation of BACE1 mRNA in mouse brain cortex after 72 h of intravenous delivery of the engineered exosomes (Alvarez-Erviti et al., 2011). Moreover, this approach proved immunocompatible as no increase in serum levels of critical pro-inflammatory cytokines TNF-α, IFN-α, IL-6, and IFN-γ-induced protein-10 were observed (Alvarez-Erviti et al., 2011). These seminal studies laid the groundwork for exosome-mediated delivery of siRNA molecules as a targeted therapeutic strategy in diverse medical conditions, including cancer, which is the focus of this review.

The following section summarizes the data from numerous studies in which engineered exosomes have been used to deliver siRNA *in vitro* and *in vivo*, targeting various cancers. These studies are summarized in Table 2.

**TABLE 2:** Summary of exosome-mediated siRNA delivery in diverse types of cancer.

Exosome source and modification (if any)	siRNA loaded	Method of exosome loading	Target cancer types	Molecular mechanisms and reported effects	References
HEK-293 T cell-derived	PD-L1 siRNA and CTLA-4 siRNA	Electroporation	Colorectal cancer	Knockdown PD-L1 and CTLA-4 gene expression. Decreased apoptosis of CD8^+^ T cells and increased their percentage in co-cultures, upregulated the expression of TNF-α, IFN-γ and IL-2. Inhibit CRC cell proliferation, repress immune escape of CRC cells, activate an anti-tumor response, and suppress tumor growth *in vivo*	Li et al. (2023)
A549 lung cancer cells. PEG-PEI-modified (Exo-PEG-PEI)	PD-L1 siRNA	Incubation	NSCLC	Knockdown of PD-L1 mRNA led to the inhibition of cell proliferation and induction of apoptosis of lung cancer cells. Reduced toxicity on normal endothelial cells	Lin et al. (2022)
Breast cancer-cell-derived	S100A4 siRNA (siS100A4)	Incubation and extrusion	TNBC	CBSA/siS100A4@Exosome accumulated in the lungs and knocked down the S100A4 gene. This led to the inhibition of the growth of lung-metastasised malignant breast cancer cells	Zhao et al. (2020)
HEK293T cell-derived. Engineering exosome (iRGD peptide-modified exosome)	CPT1A siRNA	Transfection reagent (Lipofectamine 2000)	Colon cancer	Silencing CPT1A inhibits fatty acid oxidation, thereby suppressing oxaliplatin resistance and inhibiting tumor growth	Lin et al. (2021)
HEK293T cell-derived. tLyp-1-engineered exosome	SOX2 siRNA	Electroporation	NSCLC	Effective uptake by lung cancer cells. Knockdown of the SOX2 mRNA. Decreased the stem cell population of lung cancer cells	Bai et al. (2020)
HEK293T cell-derived	c-Met siRNA	Transfection reagent (Lipofectamine 2000)	Gastric cancer	Inhibiting the expression of c-Met suppressed invasion and migration. Reversed the drug resistance of gastric cancer cells *in vitro* and significantly inhibited tumor growth *in vivo*	Zhang et al. (2020)
HEK293 and mesenchymal stem-cell-derived exosome	PLK-1 siRNA	Electroporation	Bladder cancer	Knockdown of PLK-1 mRNA and protein expression inhibits bladder cancer cell proliferation and induces apoptosis	Greco et al. (2016)
HEK293T cell-derived. Engineering exosome (DARPin G3- modified exosome)	TPD52 siRNA	Electroporation	TNBC	Binding specifically to HER2/Neu and siRNA molecules against the TPD52 gene led to the inhibition of tumor growth	Limoni et al. (2019)
HEK293T cell-derived. Engineering exosome (iRGD peptide-modified exosome)	KRAS siRNA	Transfection reagent (Lipofectamine 2000)	NSCLC	Silencing KRAS gene expression, thereby inhibiting tumor growth	Zhou et al. (2019)
Milk-derived exosomes. Folic acid-functionalized	VEGF, EGFR, AKT, MAPK, siKRASG12S. KRAS siRNA	Electroporation and exosome transfection reagent (Exo-Fect)	Lung, breast, pancreatic, and ovarian cancers	Knockdown of *KRAS* Suppressed the proliferation of A549 cells. Decreased A549 tumor xenografts. Decreased systemic toxicity in nude mice	Aqil et al. (2019)
Normal fibroblast-like mesenchymal cell-derived	KRAS G12D mutant siRNA	Electroporation	Pancreatic cancer	*KRAS*G12D knockdown and reduced phosphorylated-ERK protein levels suppressed metastasis and increased the overall survival of mice	Kamerkar et al. (2017)
HEK293T cell-derived	HGF siRNA	Transfection reagent (Lipofectamine 2000)	Gastric cancer	Reduced cell proliferation, tumor growth and angiogenesis	Zhang et al. (2018a)
Breast cancer-cell-derived exosome	MALAT1 siRNA	Transfection reagent (Lipofectamine 2000)	Breast cancer	Down-regulating the expression of MALAT1. Suppressing cell proliferation and tumor growth	Zhang et al. (2018b)
PANC1 cancer-cell-derived exosome	PAK4 siRNA	Electroporation	Pancreatic cancer	Down-regulating the expression of PAK4. Inhibiting tumor growth and increasing mice survival	Xu et al. (2021)
Human skin-derived fibroblasts (NB1RGB cells)	LCP1 siRNA	Electroporation	Oral cancer	Suppressing LCP1 expression. Suppressing the oncogenic activity of cancer cells	Kase et al. (2021)
HEK293T cells	TRPP2 siRNA	Incubation	Pharyngeal squamous cell carcinoma	Suppressing TRPP2 protein expression levels. Significantly increased E-cadherin expression and significantly decreased N-cadherin and vimentin expression, inhibiting migration, invasion and the EMT of cancer cells	Wang et al. (2019)
MCF7, MCF-7/ADR cancer-cell-derived iRGD-modified exosomes	CD44 siRNA	Electroporation	Multidrug-resistant breast cancer	Suppressing CD44 expression. Decreased cell proliferation and tumor volume and enhanced susceptibility to doxorubicin	Wang et al. (2020b)
MCF10A cells	CDK4 siRNA	Electroporation	Breast cancer	Downregulating the CDK4 mRNA and protein expression, thereby inducing G1 cell cycle arrest and inhibiting tumor growth	Yang et al. (2016)
HeLa cell-derived	RAD51 and RAD52 siRNAs	Electroporation	Cervical carcinoma	Silencing RAD51/RAD52 expression and inducing G2/M phase cell cycle arrest. Apoptosis induction of tumor cells	Shtam et al. (2013)
Bone marrow mesenchymal stem-cell-derived exosome	GRP78 siRNA	Transfection reagent (Lipofectamine 2000)	Hepatocellular carcinoma	Inhibiting the expression of GRP78, thereby suppressing the growth and invasion of cancer cells. Suppression of drug resistance	Li et al. (2018a)
Bone marrow mesenchymal stem-cell-derived exosome	Galectin-9 siRNA	Electroporation	Pancreatic ductal adenocarcinoma	Inducing tumor-suppressive macrophage polarization, cytotoxic T lymphocyte recruitment, and Tregs downregulation. Eliciting anti-tumor immunity	Zhou et al. (2021)
RAW 264.7 macrophage-derived. Engineering exosome (cRGD peptide-modified exosome)	FGL1 siRNA, TGF-β1 siRNA	Exosome transfection reagent (Exo-Fect)	Colorectal cancer	Blocking immune checkpoint FGL1 and inducing an increased number of tumor infiltration CD8 + T cells, fewer immunosuppressive cells, a significant anti-tumor effect	Pei et al. (2021)
HEK293 cells	SCD-1 siRNA	Electroporation	Anaplastic thyroid carcinoma	Regulating fatty acids metabolism and increasing ROS level. Inhibiting cellular proliferation and promoting cellular apoptosis	Wang et al. (2022)
Natural killer cells NK92MI	BCL-2 siRNA	Co-incubation	Breast cancer	Inhibiting the expression of BCL2 and enhancing cancer cells’ intrinsic apoptosis	Kaban et al. (2021)
HEK293T cell-derived.E3 aptamer-engineered exosome	SIRT6 siRNA	Electroporation	Prostate cancer	Inhibiting the expression of SIRT6 and suppressing tumor growth and metastasis	Han et al. (2021)
Ginger-derived exosomes, engineered via ligand-displaying arrow tail RNA nanoparticles	Survivin siRNA	Exosome transfection reagent (Exo-fect)	Cervical cancer	Knockdown of survivin and inhibition of tumor growth on a xenograft model	Li et al. (2018b)
HEK293T cell-derived. EGFR RNA aptamer-modified exosome	Survivin siRNA	Exosome transfection reagent (Exo-fect)	NSCLC	Suppress the expression of surviving, sensitization of cancer cells to chemotherapy, and tumor growth inhibition	Li et al. (2021)
HEK293T cell-derived engineered exosome (RNA nanotechnology-modified exosome)	Survivin siRNA	Exosome transfection reagent (Exo-fect)	Breast, prostate, and colorectal cancer	Knockdown the expression of survivin and stimulating tumor regression	Pi et al. (2018)

#### 1.7.1 Pancreatic cancer

Pancreatic cancer is an aggressive malignancy infamously resistant to chemotherapy with a dismal 5-year survival rate of 12% for metastatic disease. KRAS is a GTPase mutated in about 25% of all human cancers and sits at the crux of molecular pathways driving tumorigenesis. Specifically, KRAS is mutated in 85%–90% of pancreatic cancers and is crucial to its progression. KRAS has been viewed as a challenging therapeutic target and even dubbed as ‘undruggable’ after protracted efforts to target KRAS with small molecules have proven futile (Huang et al., 2021), making siRNA-mediated KRAS knockdown an attractive option. An interesting study by Kamerkar and colleagues showed that siRNA-loaded fibroblast-derived exosomes (iExosomes) specific to oncogenic KRAS demonstrated superior gene knockdown ability compared to siRNA-loaded liposomes. The authors further proved that this enhanced KRAS targeting was aided by macropinocytosis and reliant on CD47 (Kamerkar et al., 2017). The iExosomes demonstrated specifically repressed KRAS activity in Panc-1 cells (with a KRAS^G12D^ mutation) by inhibiting cell proliferation and augmenting apoptosis, but not in normal pancreatic epithelial cells with wild-type KRAS (BxPC-3 cells (KRAS^WT^)), MIA PaCa-2 (KRAS^G12C^) Capan-1 (KRAS^G12V^), cancer cells (Kamerkar et al., 2017). Galectin-9 is a β-galactoside-binding lectin that has been shown to promote immunosuppression by inhibiting T-cell activity, and programming regulatory macrophages (Lih et al., 2023). Serum levels of galectin-9 were proven to differentiate pancreatic ductal adenocarcinoma (PDAC) patients from benign pancreatic disease and healthy subjects (Lih et al., 2023). Researchers used bone marrow mesenchymal stem-cell-derived exosomes to deliver Galectin 9 siRNA to target pancreatic ductal adenocarcinoma (PDAC) thereby boosting immunotherapy in pancreatic cancer. These Galectin siRNA-loaded exosomes induced tumor-suppressive macrophage polarization, cytotoxic T lymphocyte recruitment, and Tregs downregulation, thereby eliciting anti-tumor immunity in orthotopic PDAC mice (Zhou et al., 2021).

#### 1.7.2 Gastric cancer

Drug resistance is the leading cause of poor prognosis of gastric cancer chemotherapy. N-methyl-N′-nitroso-guanidine human osteosarcoma transforming gene (*MET*) is a proto-oncogene that encodes a receptor tyrosine kinase, c-MET, whose activation through its natural ligand, hepatocyte growth factor, is crucial for cell morphogenesis, proliferation, migration, and protection from apoptosis (Bladt et al., 1995; Ma et al., 2003). However, aberrant expression or activation of c-MET has been implicated in several malignancies such as breast, liver, lung, colorectal, and gastric cancers, making it an attractive target for therapeutic intervention (Goyal et al., 2013; Hack et al., 2014; Ichimura et al., 1996; Zhang et al., 2020). Although targeting HGF/c-MET has improved clinical outcomes in some cancers, monotherapy targeting HGF/c-MET has been unsuccessful in proving considerable clinical efficacy in several cancers (Fu J. et al., 2021). Zhang and colleagues demonstrated that c-MET siRNA, loaded in HEK293T-derived exosomes, can reverse cisplatin resistance in gastric cancer by inhibiting migration and invasion of gastric cancer cells, promoting apoptosis *in vitro* and suppressing tumor growth in nude mice (Zhang et al., 2020). These researchers showed that c-MET expression was significantly reduced in the human gastric adenocarcinoma cell line, SGC7901, when the cells were treated with siRNA-c-MET-loaded exosomes using qPCR and Western blotting.

#### 1.7.3 Breast cancer

Breast cancer is the most frequently diagnosed cancer and the second leading cause of cancer-associated deaths in women (Libson and Lippman, 2014). The molecular mechanisms of breast cancer progression center on hormone dependence and specific gene mutations. Besides the influence of well-known molecular aberrations in breast cancer such as *BRCA1, BRCA2, HER2,* and *Ras,* other genes such as *S100A4*, *TPD52,* and *CDKs* have been identified.

S100A4, a calcium-binding protein known as metastasin-1 or fibroblast-specific protein-1 (FSP1), plays a significant role in tumor proliferation, invasion, and metastasis. S100A4 is a prognostic marker and therapeutic target in various cancers (Dahlmann et al., 2016; Liu et al., 2019; Mishra et al., 2012; Stein et al., 2006). One mechanism of S100A4-mediated promotion of carcinogenesis in colorectal cancer is its ability to be transactivated by β-catenin, an essential signaling protein in the WNT pathway (Stein et al., 2006). It is well known that the WNT-β-catenin signaling pathway is deregulated in several cancers, especially colorectal. In lung cancer cells, S100A4 knockdown lowers oxygen consumption rates, mitochondrial activity, and ATP synthesis, shifting cell metabolism to increased glycolytic activity (Liu et al., 2019). This metabolic reprogramming, characterized by enhanced glycolysis and suppressed oxidative phosphorylation to support elevated energy demands of proliferating cells and their biosynthetic activities, is an essential hallmark of cancer.

Triple-negative breast cancer (TNBC) is an aggressive phenotype of breast cancer characterized by the absence of HER-2 protein, estrogen and progesterone receptors (Dent et al., 2007). It shows poor clinical response to therapy and constitutes 15%–20% of all breast cancers (Dent et al., 2007). Research by Zhao and colleagues showed that exosome-mediated S100A4 siRNA delivery suppressed postoperative metastasis in TNBC (Zhao et al., 2020). The researchers created biomimetic nanoparticles (cationic bovine serum albumin (CBSA) conjugated siS100A4 and exosome membrane coated nanoparticles, CBSA/siS100A4@Exosome) to enhance the delivery of drugs to the pulmonary PMN. The CBSA/siS100A4@Exosomes shielded siRNA from deterioration and demonstrated high biocompatibility. Additionally, *in vivo* investigations revealed that CBSA/siS100A4@Exosome had a stronger affinity to the lungs than CBSA/siS100A4@Liposome. Moreover, CBSA/siS100A4@Exosome displayed exceptional gene knockdown properties that markedly reduced the proliferation of malignant breast cancer cells (Zhao et al., 2020). These findings show that CBSA/siS100A4@Exosomes are a viable approach to inhibit postoperative breast cancer metastasis.

Tumor protein D52 (TPD52) is a cancer-associated protein overexpressed in many cancers, including breast, lung, prostate, ovarian, and pancreatic (Tennstedt et al., 2014). It has been associated with poor prognosis in lung, prostate, and breast cancers and has been suggested to be a promising biomarker in breast and prostate cancers (Liu et al., 2007; Shehata et al., 2008; Ummanni et al., 2008). Its role in tumor promotion, invasion, and metastasis has been established *in vitro* and *in vivo* in murine animal models. A previous study showed significant apoptosis following knockdown of TPD52 in the HER2-overexpressing human breast cancer cell line (SK-BR-3) (Shehata et al., 2008).

In one study, researchers engineered exosome-generating HEK293T cells, which expressed ligands fused with exosome markers for targeted drug delivery. Modified exosomes generated from engineered HEK293T cells targeted HER2-positive breast cancer cells. A lentiviral vector-bearing Lamp2b-DARPin G3 chimeric gene was transfected into HEK293T cells. The researchers then selected cells that stably expressed the fusion protein and isolated their generated exosomes. When the modified exosomes were encapsulated with siRNA to target the TPD52 gene in SKBR3 cells, they showed a 70% knockdown of TPD52 mRNA expression (Limoni et al., 2019).

Cyclin-dependent kinases (CDKs) are serine-threonine kinase regulators of the cell cycle, whose overexpression has been associated with cell cycle deregulation and cancer progression. Researchers have generated a high yield of size-controllable exosomes by serial extrusion of non-tumorigenic epithelial MCF-10A cells via filters containing different pore sizes (Yang et al., 2016). They then loaded siRNA into the exosomes using electroporation. Moreover, the efficacy and safety of the siRNA-loaded exosomes were evaluated *in vitro* and *in vivo*. The authors showed that CDK4 siRNA-loaded exosomes were effectively endocytosed and suppressed CDK4 gene and protein expression. The loaded exosomes induced G1 cell cycle arrest, repressed cell proliferation in MCF-7 breast cancer cells, and reduced tumor growth in xenograft mouse models compared to the controls (Yang et al., 2016).

#### 1.7.4 Lung cancer

Lung cancer is the leading cause of cancer-related deaths worldwide. Sry-box 2 (SOX2) is a transcription factor essential for maintaining pluripotent embryonic and adult stem cells in numerous tissues and is vital for early mammalian development (Gontan et al., 2008). SOX2 is overexpressed in distinct types of solid tumors, including lung tumors. It has been demonstrated in mice that SOX2 overexpression results in widespread epithelial hyperplasia and, ultimately, lung carcinoma (Lu et al., 2010).

The recently discovered tLyp-1 peptide is a ligand that selectively targets neuropilin1 and 2 (NRP1 and NRP2) (Larue et al., 2023), both of which show elevated expression in several tumor types, including non-small cell lung cancer (NSCLC) (Bai et al., 2020). These target proteins are used as receptor targets in cancer drug delivery systems. Researchers used recombinant technology to engineer tLyp-1-Lam2b (lysosomal associated membrane protein 2b)-expressing exosomes loaded with siRNA targeting the SOX2 gene in NSCLC. The tLyp-1-Lam2b-siSOX2 exosomes were efficiently taken up by lung cancer cells, which led to effective knockdown of the SOX2 gene and decreased the stem cell population of lung cancer cells (CD44^+^/CD24^−^ cells) (Bai et al., 2020). Therefore, engineered tLyp-1 exosomes suggest a promising gene delivery strategy for cancer therapy.

KRAS is notorious for being difficult-to-drug in cancer therapy (Huang et al., 2021; Zhu et al., 2022). In a study aimed at targeting KRAS in lung cancer, HEK293T cells were programmed to simultaneously express KRAS siRNA and Lamp2b, an exosomal membrane protein, coupled with a tumor-homing internalizing RGD (iRGD) peptide (Zhou et al., 2019). The data showed that iRGD-engineered exosomes specifically delivered KRAS siRNA to lung cancer cells following intravenous administration. Also, tumor growth in xenograft mouse models was suppressed by KRAS siRNA encapsulated in iRGD-exosomes (Zhou et al., 2019).

#### 1.7.5 Multiple myeloma

Multiple myeloma is a hematologic malignancy marked by aggressive plasma cell proliferation, preceded by aberrant molecular signaling, including MYC pathway dysregulation (Jovanović et al., 2018). The MYC family of proteins regulates diverse biological processes, including metabolism, apoptosis, differentiation, cell proliferation, ribosome biogenesis, and protein translation. MYC is an oncogene that is dysregulated or overexpressed in several cancers (Dang, 2012; Dang et al., 2008; Gabay et al., 2014; Soucek and Evan, 2010). The role of MYC has been established in the pathogenesis of multiple myeloma, given that its suppression has been shown to inhibit cell proliferation in multiple myeloma (Kuehl and Bergsagel, 2012).

Aberrant WNT/β-catenin signaling is critical in MM carcinogenesis and is proposed as a potential therapeutic target (van Andel et al., 2019). Pharmacological and genetic inhibition of the WNT/β-catenin pathway in multiple myeloma mouse models have shown efficacy in slowing disease progression (Ashihara et al., 2009; Yao et al., 2011).

In line with the above studies, Soma and colleagues engineered exosome-loaded antibody-siRNA complexes to repress MYC and β-catenin as a potential targeted therapeutic strategy in multiple myeloma. The researchers conjugated anti-CD63 monoclonal antibodies with siRNA using arginine linkers to target the mRNA transcripts of MYC and CTNNB1 genes in human multiple myeloma cells (OPM-2). They showed the successful uptake of the exosomes by OPM-2 cells and a corresponding decrease in mRNA transcripts of MYC and CTNNB1 to 52.5% and 55.3%, respectively (Soma et al., 2022).

#### 1.7.6 Esophageal and oral cancers

Drug resistance is the primary cause of poor clinical outcomes in patients suffering from esophageal and oral cancers. Transient receptor potential polycystic 2 (TRPP2) is a membrane-bound cation channel protein that regulates calcium homeostasis in renal epithelial cells. The expression of TRPP2 was shown to be markedly upregulated in laryngeal squamous cell and head-and-neck cancers and is involved in promoting metastasis through the EMT process (Wang et al., 2019; Wu et al., 2016). Using fluorescence microscopy and Western blotting, Wang and colleagues showed that HEK293-generated exosomes were effectively taken up by FADU (head-and-neck carcinoma) cells, and the siRNA-exosome complex significantly downregulated TRPP2 protein levels. siRNA-mediated knockdown of TRPP2 induced downregulation of N-cadherin and vimentin and increased the expression of E-cadherin in laryngeal squamous cell carcinoma cells, thereby suppressing cell migration and invasion of FADU cells (Wang et al., 2019).

Lymphocyte cytoplasmic protein 1 (LCP1) is an actin-binding protein implicated in the progression of several non-hematopoietic tumors such as prostate, breast, and oral squamous cell carcinoma (OSCC). Koide and colleagues showed that LCP1 is upregulated in OSCC, and overexpression of LCP1 correlated with tumor size and lymph node metastasis in OSCC clinical samples (Koide et al., 2017). The authors demonstrated that LCP1 knockdown suppressed the proliferation, invasion, and migration of OSCC. A recent study capitalized on this discovery by engineering normal skin fibroblast-derived exosomes to deliver siRNAs against the LCP1 gene to OSCC cells to repress the proliferation of oral cancer cells (Kase et al., 2021). The researchers used electroporation to load the siRNA into Epstein-Barr virus induced-3 (EBI3) cDNA-transfected skin fibroblast cells and isolated the exosomes by ultracentrifugation. Their data showed that isolated exosomes were stable and effective in transfecting LCP1 siRNA (siLCP1) into OSCC cells. Exosome-mediated siLCP1 delivery resulted in the downregulation of LCP1 in OSCC cells compared with control cells, triggering significant *in vitro* and *in vivo* tumor suppression (Kase et al., 2021).

#### 1.7.7 Cervical cancer

Survivin, also known as BIRC5 (baculoviral inhibitor of apoptosis repeat-containing 5), is the smallest member of the inhibitor of apoptosis (IAP) family of proteins, which participates in inhibiting apoptosis and promoting cell cycle progression (Ambrosini et al., 1997; Garg et al., 2016). Survivin has been targeted using exosome-delivered siRNAs as a therapeutic strategy in cervical cancer. Targeting survivin represents a promising anticancer therapeutic strategy since survivin is mutated or overexpressed in many malignancies but not in normal, fully differentiated cells, making it a promising biomarker (Ambrosini et al., 1997). Overexpression of survivin is linked to chemoresistance, higher tumor relapse, and poor patient prognosis (Altieri, 2003).

In one innovative study, survivin siRNA loaded in folate-decorated exosomes demonstrated superior targeted siRNA delivery to tumors and enhanced the siRNA efficacy compared to folate-conjugated siRNA controls. Since endosomal trapping is one of the challenges faced by siRNA molecules, the researchers decorated HEK293T-derived exosomes with folate, a surface glycoprotein receptor, to increase its targeting efficiency (Zheng et al., 2019). The researchers demonstrated that the folate ligand displayed on the exosome surface promoted the targeted delivery to tumors and improved siRNA-mediated gene knockdown, leading to efficient tumor suppression of cervical cancer (Zheng et al., 2019).

#### 1.7.8 Bladder cancer

The Polo-like kinase-1 (PLK-1) gene influences mitotic progression, promoting mitotic entry, segregation of sister chromatids, spindle formation, and cytokinesis. PLK-1 expression is upregulated in a variety of cancer types, including bladder cancer, and its overexpression is linked to poor prognosis, relapse, and metastasis (Nogawa et al., 2005). Moreover, PLK1 has been identified as a prognostic biomarker in non-muscle invasive bladder cancer (Fristrup et al., 2012), further supporting its relevance in tumor progression and targeted therapy. Several studies have demonstrated that the knockdown of PLK-1 induces cell cycle arrest and apoptosis in bladder cancer cells (Fristrup et al., 2012; Nogawa et al., 2005; Seth et al., 2011). Moreover, two lipid nanoparticle formulations delivering PLK-1 siRNA are currently undergoing clinical trials (Table 1). In one study, exosomes were isolated from HEK293 and mesenchymal stem cells by ultracentrifugation and used to deliver PLK-1 siRNA to bladder cancer cells. The authors showed that the PLK-1 siRNA-loaded mesenchymal stem cell-derived exosomes induced knockdown of PLK-1 mRNA and protein expression, induced apoptosis, and inhibited bladder cancer cell proliferation (Greco et al., 2016).

#### 1.7.9 Colorectal cancer

Immunotherapeutic strategies are currently being pursued in colorectal cancer treatment. Programmed cell death protein 1 (PD-1) can regulate the activation of T-cells after binding to its cognate ligand programmed cell death ligand 1. This activation suppresses cytokine expression of TNF-alpha and IFN-gamma and inhibits T-cell proliferation. Immune checkpoint proteins, such as PD-L1/PD-L2, are known to interact with their cognate ligand, PD-1, to promote evasion of immunosurveillance by malignant cells by neutralizing T-cell activation signals. Inhibiting immune checkpoint activates cytotoxic T cells, thereby enabling them to recognize cancer-associated antigens and reactivate anti-tumor immune responses (Han et al., 2020; Pascual et al., 2019; Tumeh et al., 2014).

PD-L1 expression is frequently expressed in activated T cells and human malignancies. Expressed PD-L1 on cancer cells can bind to PD-1 receptors on the surface of tumor-infiltrating T-lymphocytes, preventing T-cell activation and eventually leading to tumor immune escape (Tumeh et al., 2014). Due to the relevance of immune checkpoints in cancer progression, immune checkpoint inhibitors for PD-1 and PD-L1 have been approved for certain types of tumors. Similarly, CTLA-4 has been considered a marker for immunosurveillance in colorectal cancers. This has prompted the exploration of anti-CTLA-4 antibodies as immunomodulatory and immunotherapeutic strategies in colorectal carcinoma (Li et al., 2023; Narayanan et al., 2022). It has been observed that CTLA-4 and PD-L1 expressions are high in colorectal cancer cells. In one study, exosomes loaded with PD-L1, and CTLA-4 siRNAs were shown to knock down PD-L1 and CTLA4 gene expression, inhibiting colorectal cancer cell proliferation and tumor growth *in vivo*. The engineered exosomes containing PD-L1, and CTLA-4 siRNA activated tumor immune response *in vivo* and repressed their immune escape of colorectal cancer cells. Their findings demonstrated that PD-L1 and CTLA-4 siRNA-loaded exosomes could suppress colorectal cancer progression and improve tumor immune responses (Li et al., 2023).

Fatty acid oxidation (FAO) is a metabolic process critically involved in chemoresistance and cancer progression, making it an emerging therapeutic target in cancer (Lin et al., 2021; Qu et al., 2016). Carnitine palmitoyltransferase 1A (CPT1A) is an essential enzyme in FAO whose overexpression has been reported in colon cancer cells and tissues (Lin et al., 2021). High expression of CPT1A has been correlated with resistance to oxaliplatin, while low expression was observed in oxaliplatin-sensitive colorectal cancer cells (Lin et al., 2021). Lin et al. (2021) engineered iRGD-modified exosomes and encapsulated them with siRNA targeting CPT1A in colorectal cancer cells leading to CPT1A knockdown, suppressing FAO and reversing oxaliplatin resistance. iRGD-modified exosomes significantly suppressed the expression of CPT1A in tumor tissues, reversed oxaliplatin resistance, and inhibited tumor growth by inhibiting FAO with high safety *in vivo*.

Exosomes can be flexibly engineered to display peptide ligands, enhancing their targeting specificity and uptake efficiency. Internalizing arginine-glycine-aspartic acid (iRGD) is a 9-amino acid cyclic peptide (sequence: CRGDKGPDC) that can be displayed on exosomes to target exosomes to cancer cells (Lin et al., 2021; Zhou et al., 2019; Zuo, 2019). The fusion of iRGD with exosomal surface proteins such as Lamp2b results in the successful display of iRGD on exosomes. iRGD binds with high specificity to the avβ3 and avβ5 integrins, which are highly expressed on tumor vascular epithelial cells and tumor cells (Tian et al., 2014). In a recent study to suppress diffuse large B-cell lymphoma (DLBCL) progression, researchers engineered iRGD-modified exosomes to deliver B-cell lymphoma 6 (BCL6) siRNA to knock down BCL6. iRGD-Exo-siRNA complex suppressed the proliferation of DLBCL cells *in vitro* and inhibited tumor growth in DLBCL *in vivo* with minimal toxicity in mice (Liu et al., 2022). These data suggest a strong therapeutic relevance of iRGD-engineered exosomes as a delivery strategy for RNAi in DLBCL.

### 1.8 Advanced siRNA modifications and *in vivo* self-assembly of siRNA in cancer therapy

A variety of delivery vehicles, such as cationic polymers, viruses, and lipid nanoparticles, and conjugated ligands, such as trivalent N-acetylgalactosamine (GalNAc), have been developed to improve the efficiency of siRNA delivery *in vivo* (Isazadeh et al., 2023; Nair et al., 2014; Singh et al., 2018). Studies have developed a chemical modification strategy that employs covalent conjugation of a synthetic trivalent N-acetylgalactosamine ligand to modify siRNA chemically. Glycoproteins with terminal GalNAc sugars have a high affinity and specificity to asialoglycoprotein, a receptor that is abundantly expressed in hepatocytes. This binding triggers the uptake of functionalized moieties such as siRNA by hepatocytes, as previously described (Nair et al., 2014). This method has now been harnessed to deliver several RNAi-based therapeutics in pre-clinical models and clinical trials (Butler et al., 2013; Chan et al., 2015; Huang, 2017).

A new epoch in siRNA-based therapeutics was launched with the FDA’s approval of the first siRNA-lipid nanoparticle complex drug, Patisiran, for the treatment of polyneuropathy of hereditary transthyretin-mediated amyloidosis (Hoy, 2018); and Givosiran, a siRNA-conjugated GalNAc ligand that enables asialoglycoprotein receptors-mediated targeted delivery to hepatocytes to treat acute hepatic porphyria (Honor et al., 2021); and lumasiran, a siRNA-conjugated GalNAc ligand for the treatment of primary hyperoxaluria type 1 (PH1) (Scott and Keam, 2021). Although siRNA-conjugated GalNAc formulations have not been approved for cancer treatment, recent studies have shown the efficacy of these formulations to target hepatocellular carcinoma in xenograft models (Neumayer et al., 2024).

siRNAs are commonly coupled with ligands or loaded into vehicles *in vitro*. Nevertheless, when delivered *in vivo*, these pre-assembled siRNA complexes are usually fraught with challenges such as poor stability in circulation, high toxicity, low immunocompatibility, and tissue delivery (Yu et al., 2023).

**FIGURE 5:**
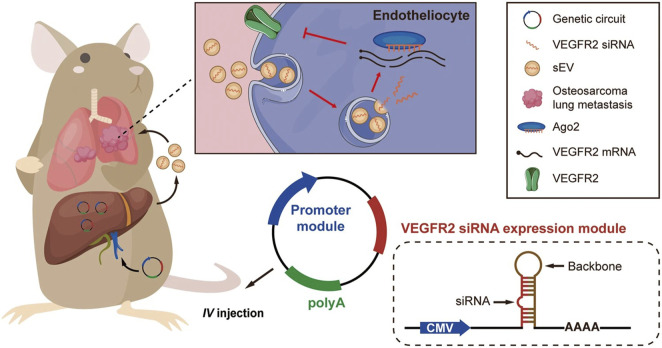
*In vivo* delivery of an siRNA-expressing genetic circuit. The genetic circuit is comprised of a VEGFR2 siRNA-expressing backbone and a CMV promoter. Following intravenous administration, the anti-VEGFR2 circuit is transported to the liver. In hepatocytes, the CMV promoter controls the transcription of VEGFR2 siRNA and enables the encapsulation of VEGFR2 siRNA into sEVs. After being released into the blood circulation, VEGFR2 siRNA-loaded sEVs then accumulate metastatic lesions of the lungs, such as vascular endothelial cells. Lastly, VEGFR2 mRNA is knocked down, and the angiogenesis is suppressed, thereby inhibiting osteosarcoma lung metastasis. The siRNA-expressing backbone could contain siRNA sequences targeting any other disease-associated gene (Figure used with permission from (Yu et al., 2023)).

Despite the success achieved in RNAi modification strategies, efficient *in vivo* siRNA delivery remains the most challenging limitation for widespread clinical translation of siRNA-based therapeutics. To circumvent this limitation, recent studies have developed plasmid-based genetic circuits that can reprogram mouse host liver cells to synthesize siRNAs and stimulate their self-assembly into secretory exosomes thereby promoting the *in vivo* siRNA (Fu Z. et al., 2021; Yu et al., 2023). In one of the studies, the genetic circuit was composed of two parts: a siRNA-expressing backbone that encoded a VEGFR2-targeting siRNA, and a cytomegalovirus (CMV) promoter that controlled the expression of VEGFR2 siRNA (Figure 5). In a similar study, the circuit was designed to contain two siRNA-expressing backbones to knock down cancer-associated genes simultaneously (Fu J. et al., 2021). These studies showed that when the circuit is delivered into the animal, it is transported to liver cells where the circuit’s promoter drives the transcription of the siRNA and conveys the siRNA into exosomes (Fu Z. et al., 2021; Yu et al., 2023).

Angiogenesis, a critical driver of solid tumor growth, is controlled by the vascular endothelial growth factor (VEGF-A) and its receptors, including VEGFR1, VEGFR2, and VEGFR3 (Moens et al., 2014). VEGF-A, which is highly expressed on tumor cells, binds VEGFR2 on endothelial cells, thus leading to angiogenesis (Carmeliet, 2000). Targeting VEGFR2 has been proven to be a viable anticancer strategy (Liu et al., 2023). Osteosarcoma is the most prevalent and highly metastatic primary malignant bone tumor in most countries (Marko et al., 2016). A research study demonstrated enhanced expression of VEGFR2 and increased sprouting of new blood vessels in lung metastatic osteosarcoma specimens. Inspired by these data, the researchers engineered a self-assembled VEGFR2 siRNA intravenously delivered to mice. They proved that the knockdown of VEGFR2 with *in vivo* self-assembled VEGFR2 siRNA suppressed osteosarcoma lung metastasis. Control mice were treated with Apatinib, a VEGFR2-specific tyrosine kinase inhibitor approved for osteosarcoma treatment (Liu et al., 2021). The results proved that mice treated with the anti-VEGFR2 circuit exhibited longer survival times than the control mice, with 30% surviving for more than 80 days post-treatment (Yu et al., 2023). These data indicate that targeting VEGFR2 with exosome-mediated delivery of VEGFR2 siRNA could be a viable therapeutic strategy for treating lung metastatic osteosarcoma.

### 1.9 siRNA delivery challenges in cancer

Loading siRNA into exosomes is another challenging feat in exosome-mediated siRNA delivery. Several methods, including incubation (Didiot et al., 2016), sonication (Lamichhane et al., 2016), transfection using reagents (Zhao et al., 2020), and electroporation have been used to load small RNAs into exosomes (Didiot et al., 2016; Faruqu et al., 2018; Lamichhane et al., 2016; Limoni et al., 2019; Zhao et al., 2020). The most frequently used method is electroporation. Although experiments have established that loading siRNAs into exosomes by electroporation can efficiently induce *in vitro* and *in vivo* silencing, multiple papers have noted difficulties using this technology because of significant variability. Kooijmans et al. (2013) showed that electroporation could stimulate siRNA precipitation and aggregation, which could over-estimate loading exosomes with siRNA (Kooijmans et al., 2013). To mitigate this effect, pioneering work by Byrne et al. (2013) showed that hydrophobic modification of siRNA by conjugating a cholesterol moiety to the 3′ end of the passenger strand improves siRNA stability and enhances its cellular internalization with a significant reduction in target mRNA expression (Byrne et al., 2013).

Extracting exosomes from diverse origins and non-standard exosome purification methods may constitute a critical challenge for the clinical translation of exosome-based therapies. Some studies have even reported varied efficiencies of drug delivery associated with diverse types of exosomes. For example, Melzer et al. (2019) showed that mesenchymal stem cell (MSC)-derived exosomes were more efficient drug delivery vehicles, leading to higher cytotoxicity in several types of cancer cells than HuVEC-derived exosomes (Melzer et al., 2019). Moreover, it has been demonstrated that MSC-derived exosomes exhibit better tumor targeting and accumulation than exosomes generated from A431 tumors (Cohen et al., 2021). Interestingly, the A431-derived exosomes showed a higher drug loading capacity than the MSC-derived exosomes (Cohen et al., 2021). The choice of exosomes used will therefore influence their therapeutic benefit. Hence, careful consideration in line with desired exosome characteristics is required for therapy.

Numerous studies are also exploring the therapeutic advantages of plant-derived exosomes (Dad et al., 2021; Mu et al., 2023).

### 1.10 Plant-derived exosomes: unlocking the potential of nature’s nanoscale messengers

Pharmaceutical research has long been at the forefront of exploring innovative methods to enhance the delivery of active ingredients in medications. The goal has always been to improve efficacy while minimizing the undesirable side effects often associated with chemical or biological products (Fais et al., 2016; Yáñez-Mó et al., 2015). To this end, various synthetic nanoparticles including polymeric nanoparticles, solid lipid nanoparticles (SLNs), crystal nanoparticles, and liposomes have been developed. These nanoparticles serve as delivery vehicles, encapsulating therapeutic molecules and shielding them from degradation until they reach their intended target sites. Each type of nanoparticle exhibits unique advantages and disadvantages as drug delivery vehicles (DDVs), with the capability of encapsulating both hydrophilic (such as siRNA, RNA, and DNA) and hydrophobic (including proteins, peptides, and antibodies) bioactives. However, the clinical application of these synthetic systems is hindered by the need for extensive *in-vivo* toxicity evaluations and the high costs associated with their production (Federici et al., 2014; Orefice et al., 2023; Sivadasan et al., 2021).

As a potential solution to these challenges, plant-derived exosome-like nanoparticles (PDENs) emerge as a promising nanomedicine tool. PDENs ranging in size from micro to nanometers, can be sourced organically, offering a safer and more cost-effective alternative for drug delivery in clinical applications (Barzin et al., 2023; Sarasati et al., 2023; Sarvarian et al., 2022; Zhao et al., 2023). PDENs are emerging as promising candidates for overcoming the technical challenges associated with human cell-derived exosomes (Sall and Flaviu, 2023). Researchers are actively exploring the potential of these nanoparticles for large-scale production, disease therapy, and the generation of nanoparticle DDVs. They exhibit favorable physiological, chemical, and biological characteristics that enhance their usability across various medical applications (Dhar et al., 2024; Madhan et al., 2024; Rome, 2019; Yang et al., 2018).

Despite their similarities, PDENs and human cell-derived exosomes exhibit notable differences, particularly in lipid composition and biological functions. Notably, they offer several advantages over mammalian exosomes, such as reduced immunogenicity, improved bioavailability, large-scale production, and a remarkable safety profile (Dad et al., 2021; Mu et al., 2023). While PDENs have been known for nearly 60 years, interest in their potential has only recently surged, highlighting a significant gap in research that is now being actively addressed. Studies have shown that PDENs possess superior bioavailability to miRNAs in either free form or when bound to proteins, facilitating more effective therapeutic applications (Sundaram, 2019). Moreover, PDENs demonstrate exceptional stability within the gastrointestinal tract, making them versatile for oral or intranasal administration (Ju et al., 2013; Teng et al., 2018). Compared to conventional natural products, PDENs exhibit targeted delivery to specific organs and display enhanced solubility, greater permeation across biological barriers, and quicker dissolution into the bloodstream, all while minimizing potential side effects.

Recent literature highlights the promising application of PDENs to treat periodontitis, primarily through their ability to inhibit inflammation and combat periodontal pathogens (Sundaram et al., 2019; Zhang Y. et al., 2022). A notable study by Wang and colleagues introduced multifaceted PDENs as nanovectors designed for the targeted delivery of therapeutic agents to brain tumors. They demonstrated that these PDENs not only accumulated in specific tissues but also maintained long-term circulation in peripheral blood due to their remarkable stability (Wang et al., 2013). Moreover, an analysis of various plants revealed significant quantities of PDENs, with measurements of 1.76 mg/g in grape, 2.21 mg/g in grapefruit, and 0.44 mg/g in tomato, which indicates that these plants could facilitate large-scale production of PDENs. Furthermore, ginger-derived exosomes (GDEs) have exhibited the capacity to suppress tumor cell proliferation and alleviate inflammatory bowel disease (IBD) by effectively targeting and regulating gut microbiota following tissue damage (Teng et al., 2018; Zhang et al., 2016; Zhang et al., 2017). In another study, engineered GDEs efficiently delivered survivin siRNA in cervical cancer cell lines and mouse xenograft tumor models. This led to effective knockdown of survivin and inhibition of tumor growth. The GDEs also exhibited high biocompatibility and low toxicity, as evidenced by insignificant treatment-associated body weight changes in the mice (Li H. et al., 2018). Importantly, investigations into the safety profile of PDENs have indicated a lack of associated inflammation or toxicity, contrasting with traditional artificial nanocarriers made from copolymers, metals, or carbon (Kim et al., 2022; Wolfram et al., 2015). This positions PDENs as a safer alternative for nanocarrier applications. Furthermore, several preclinical studies involving both human and plant-derived nanovesicles provide a robust foundation for future clinical trials, demonstrating the potential of natural nanovesicles in drug delivery (Orefice et al., 2023).

Collectively, these findings offer significant hope for enhancing efficacy and minimizing the toxicity of existing and newly developed therapeutic compounds in treating various diseases. This combination of properties positions PDENs as a multifaceted tool in modern therapeutic applications, paving the way for innovative advancements in drug delivery and treatment strategies. As ongoing research unravels their potential, harnessing these natural nanocarriers could revolutionize therapeutic delivery systems and plant science, promoting sustainable practices and improving human health.

### 1.11 Conclusion and future directions

Gene therapy involves the delivery of nucleic acids to specific target cells for diseases previously deemed challenging to treat with conventional medication. Developing an optimal delivery system for gene therapy that efficiently transports nucleic acid cargo while minimizing the side effects of nucleic acid cargo has proven to be a significant hurdle. Various viral and non-viral vectors have been developed, yet clinical success remains limited due to associated challenges. Consequently, there is an ongoing need to develop safe and efficient delivery systems. Using cell secretory nano-vesicles, particularly exosomes, for nucleic acid delivery has sparked excitement in gene therapy, which is likely attributed to their extended plasma half-life, biocompatibility, favorable pharmacokinetics, minimal toxicity, and the ability to traverse biological barriers (Akbari et al., 2022; Banerjee and Rajeswari, 2023; Kar et al., 2023; Meng et al., 2020; Wu et al., 2017; Zeng et al., 2022). To date, researchers have employed two main approaches: engineering parent cells to secrete exosomes loaded with the desired cargo, such as siRNA and mRNA, or modifying exosomes post-secretion to encapsulate the intended payload through physical or chemical means. Despite considerable progress, there are still challenges that impede the therapeutic application of exosomes.

The primary hurdle for exosome-mediated gene delivery lies in the isolation, purification, and subsequent incorporation of nucleic acids into these vesicles. Current techniques for isolating exosomes include size-based isolation, ultracentrifugation, precipitation, and microfluidic devices. However, these methods have significant drawbacks, such as being time-consuming and necessitating sophisticated equipment (Yang and Wu, 2018; Zeng et al., 2022). Various technologies, such as bioreactors, 3D scaffolds, and microfluidic devices, have been implemented to enhance the production of exosomes. For instance, Haraszti and colleagues used 3D culture and tangential flow filtration (TFF) to achieve a 140-fold increase in exosome production compared to traditional 2D or 3D cultures or TFF alone (Haraszti et al., 2018).

Additionally, studies have indicated that stress environments such as hypoxia, low pH, and exposure to anticancer drugs can stimulate the production of exosomes (Harmati et al., 2017; Kanemoto et al., 2016; King et al., 2012). Yang et al. (2020) developed a cellular nanoporation (CNP) method, producing up to 50-fold more exosomes than conventional strategies like bulk electroporation and Lipo2000 transfection. Another study demonstrated a 40-fold increase in exosome yield using a hollow fiber bioreactor (Watson et al., 2016). Furthermore, microfluidic devices have shown promise in separating and purifying exosomes. Wang and colleagues reported that a 3D nanostructured microfluidic chip captured 90% of exosomes effectively (Kanwar et al., 2014; Wang et al., 2017). Moreover, food-derived exosomes, including those from bovine milk and grapes, have demonstrated encouraging results in preclinical studies (Munagala et al., 2016; Zhang Z. et al., 2022). However, ensuring quality control while increasing exosome output is crucial, especially regarding contamination or size overlap with other EVs.

Another challenge involves developing new methods to improve the low loading efficiency of current exosome-nucleic acid-loading strategies, including incubation, electroporation, and transfection, thereby addressing the limitations of traditional techniques (Ortega et al., 2020). Several novel nucleic acid loading methods have recently been established to overcome these challenges. Li et al. (2019) developed a CD9-HuR fusion protein, which selectively enriches target RNA into exosomes, demonstrating a sevenfold increase in specific RNA miRNA155 compared to control groups (Li et al., 2019). Other approaches, such as a physical-chemical hybrid platform involving cationic LNPs exposed to cyclic stretch and thermostable ionizable lipid-like nanoparticles, offer efficient delivery of siRNA into exosomes, providing innovative strategies for overcoming limitations in gene therapy (Hu et al., 2022; Wang et al., 2021).

The next challenge comes in achieving precise, personalized treatment for cancer patients, which is hindered by the heterogeneity of exosomes and the complex *in vivo* environment, limiting precise delivery, and expected efficacy. A few studies showed that autologous exosomes could be obtained using minimally invasive techniques or surgical samples and expanded *in vitro* under specific culture conditions to use as efficient delivery carriers, offering remarkable targeting ability against cancer cells (Gong et al., 2021; Wang et al., 2021). Given that exosomes contain diverse proteins and functional immune cells, their use could elicit a robust immune response from the host, potentially resulting in rapid clearance. Consequently, performing a thorough preclinical evaluation encompassing pharmacokinetics, toxicity profiles, and pharmacodynamics analyses becomes imperative to mitigate potential adverse effects. However, the characterization and structural identification of exosomal proteins would offer valuable insights for efficient anticancer drug development, potentially using exosomes loaded with siRNAs to target these proteins as a promising strategy for targeted anticancer therapy.

Recently, researchers have been focusing increasingly on exploring the isolation of exosomes from various plant sources as a potential solution for drug delivery systems to treat cancer (Di Gioia et al., 2020; Feng et al., 2024; Li Z. et al., 2018; Madhan et al., 2024; Sall and Flaviu, 2023). Plant-derived exosomes offer several advantages over cancer cell-derived exosomes: they are naturally provided and play roles in intercellular communication, possess phospholipid-rich characteristics that protect cargo, use natural mechanisms for cellular uptake, are immune-tolerant due to their presence in human ingested foods, demonstrate scalability for industrial use, are non-toxic as they originate from organic sources, and are readily available from various plant types that can be cultivated (Sarasati et al., 2023; Yi et al., 2023).

Despite extensive research on plant-derived exosome-like nanoparticles, current knowledge regarding their manufacturing processes, biological mechanisms, and applications remains limited. This gap presents significant opportunities for further research, development, and translational efforts. A deeper understanding of plant-derived exosomes in the future could potentially revolutionize natural medicine, offering compounds that are abundantly available, more effective, efficient, and associated with significantly fewer adverse effects compared to currently available medications. Despite significant challenges and difficulties, exosome-based siRNA delivery systems retain tremendous potential as the next-generation of nanomaterials for advanced drug delivery and cancer treatment.
